# Dyselectrolytemia in Children With Severe Pneumonia: A Prospective Study

**DOI:** 10.7759/cureus.53940

**Published:** 2024-02-09

**Authors:** Vineeta Pande, Renuka Jadhav, Md Ilyaz, Shailaja Mane, Jasleen Dua

**Affiliations:** 1 Pediatrics, Dr. D. Y. Patil Medical College, Hospital, and Research Centre, Dr. D. Y. Patil Vidyapeeth, Pune, IND

**Keywords:** hyperkalemia, hypokalemia, hypernatremia, hyponatremia, dyselectrolytemia, pneumonia

## Abstract

Background

Pneumonia is a condition characterized by inflammation of the lung parenchyma. It is one of the leading causes of mortality in children below five years of age. While predominantly prevalent in developing countries, it is also associated with significant healthcare-associated costs in developed countries. Among the many risk factors for childhood pneumonia, incomplete immunization, nonexclusive breastfeeding for less than six months, delayed weaning, poor household air quality, malnutrition, and low birth weight are the most commonly found.

Electrolyte disturbances, also known as dyselectrolytemia, have been associated with a broad spectrum of acute infections, including pneumonia, particularly hyponatremia. It occurs in the majority of community-acquired pneumonia. Hyper- and hypokalemia are less frequently occurring electrolyte disturbances. Electrolyte disturbances are due to impairment of the intrarenal mechanism of urine dilution due to extracellular fluid volume depletion and inappropriate secretion of antidiuretic hormone. The central nervous system is imminently affected by acute hyponatremia. This condition frequently culminates in cerebral edema, a result of the rapid fluid shift, and causes sudden fatality.

Aim of the study

This study aims to study dyselectrolytemia in children with severe pneumonia.

Objectives

The study objectives are to assess dyselectrolytemia in children with severe pneumonia and to correlate dyselectrolytemia with morbidity and hospital stay.

Methodology

This prospective study was conducted on 80 children in the age group of two months up to five years who visited our tertiary care center and had severe pneumonia. We evaluated the extent of dyselectrolytemia in our study population by analyzing the frequency correlation of different kinds of electrolyte imbalances. We also analyzed the correlation between morbidity and hospital stay.

Results

Out of 80 children in this study with severe pneumonia, 47 (59%) had electrolyte imbalance. Among the patients with electrolyte imbalance, 31 (39%) patients had hyponatremia followed by hypokalemia in 12 (15%) patients, hypernatremia in 3 (4%) patients, and hyperkalemia in 1 (1%) patient. Among the 17 (21%) children with pneumonia requiring ICU admission, 16 (94%) had dyselectrolytemia and 4 (24%) experienced fatal outcomes.

Conclusions

The majority of the children who were admitted to the ICU had severe pneumonia along with electrolyte imbalance. This necessitates the monitoring of the electrolyte and nutritional status of the patients with pneumonia. Providing proper nutrition advice for children with pneumonia may reduce morbidity and mortality. Early detection and treatment of electrolyte imbalances in pneumonia cases can decrease prolonged hospital stays, ICU admissions, and the need for mechanical ventilation, ultimately contributing to a reduction in morbidity and mortality.

## Introduction

Pneumonia is a condition characterized by inflammation of the lung parenchyma. It is reported to be the leading cause of morbidity and mortality in children less than five years of age [1]. In India, being a developing country, 13% to 16% of pediatric mortality is due to pneumonia [2]. The most prevalent clinical manifestation of pneumonia is tachypnea. Other manifestations include increased work of breathing along with nasal flaring, suprasternal, subcostal, and intercostal retractions. Severe infection is associated with cyanosis and lethargy, especially in infants. On auscultation, wheezing and crackles can be appreciated.

Childhood community-acquired pneumonia (CAP) accounts for 30% to 40% of the total pediatric hospital admissions with a mortality rate of 15% to 28% [3]. In developing countries with limited healthcare access and inadequate nutrition, lower respiratory tract infections, including atypical pneumonia, bronchiolitis, pneumonia, and SARS, pose a constant threat to the health of children. Children below five years of age, those who are unimmunized, have low birth weight, are malnourished, non-exclusively breastfed, live in overcrowded conditions, belong to a lower socioeconomic status, or are immunocompromised are recognized as major risk factors for pneumonia [4].

The Integrated Management of Neonatal and Childhood Illness (IMNCI) control program has classified pneumonia in the age group of two months to five years into two types. Severe pneumonia, characterized by lower chest indrawing, may include fast breathing, with or without additional signs such as nasal flaring, grunting, and cyanosis. Very severe pneumonia is characterized by several indicators, including the inability to drink, cyanosis, stridor in a calm child, severe respiratory distress (head nodding) or grunting, lethargy/excessive drowsiness, and convulsions [5].

The majority of pneumonia cases also exhibit electrolyte disturbances, with a higher frequency of hyponatremia associated with severe and worsening outcomes, necessitating prompt management. Dyselectrolytemia has been associated with a wide range of acute infections, including pneumonia, especially hyponatremia [6]. Hyponatremia, occurring in the majority of patients with CAP is associated with greater disease severity and worsened outcomes. Hypo- and hyperkalemia are less frequently occurring electrolyte disturbances observed in pneumonia [7]. These dyselectrolytemias often complicate the management and prognosis of pneumonia.

The previous studies suggest that electrolyte disturbances in pneumonia are due to the impairment of the intrarenal mechanism of urine dilution due to extracellular fluid volume depletion and inappropriate secretion of antidiuretic hormone (ADH) [8]. One of the most frequent electrolyte disorders, hyponatremia, is found in about 3% of hospitalized patients. Although hyponatremia is simple to identify and rarely adverse, the origin is sometimes difficult to determine, and improper fluid administration might result in associated complications. Several studies and investigations have attributed the Syndrome of Inappropriate Antidiuretic Hormone Secretion (SIADH) as the most likely cause of hyponatremia in respiratory tract infections [9].

Potassium also plays a major role in regulating the bioelectric process. Abnormality in potassium homeostasis affects bioelectric processes in cells and may lead to potentially life-threatening events like cardiac arrhythmias, muscular paralysis, cardiac arrest, respiratory failure, and paralytic ileus. Imbalance in potassium levels, which include hypo- and hyperkalemia, are other less likely occurring electrolyte imbalances with increased risk of mortality seen in pneumonia. It is mostly associated with inappropriately high circulating levels of ADH [10]. ADH promotes the secretion of potassium through distal tubules by a mechanism independent of urine flow.

Electrolyte imbalances often go undetected for a significant period, leading to substantial morbidity and mortality. The consequences may be extremely serious if serum electrolyte imbalances remain undetected and untreated. Fluid and electrolyte imbalances should be treated to regain normal physiologic homeostasis. To regain normal physiology, a high index of suspicion and timely recognition are necessary.

## Materials and methods

This is a hospital-based prospective study, with a sample size of 80 patients with pneumonia admitted to a tertiary care center. The study was approved by the Institutional Ethics Sub-Committee under protocol number I.E.S.C./C-28/15. Data about clinical, physical, and demographic parameters were collected from the patients after obtaining written consent from their parents/guardians. Children aged between two months and five years were diagnosed with pneumonia and severe pneumonia according to the World Health Organization (WHO) and Integrated Management of Neonatal and Childhood Illness (IMNCI) guidelines. Children with associated congenital heart diseases, central nervous system (CNS) infections, and those on medications that can cause electrolyte imbalance, such as diuretics and anticonvulsants, were excluded from the study. Dyselectrolytemia was defined based on globally accepted critical values.

A range of 135 to 145 mmol/L for serum sodium was considered normal. Values less than 135 mmol/L were classified as hyponatremia, while values greater than 145 mmol/L were categorized as hypernatremia. Serum potassium value in the 3.6-5.5 mmol/L range was considered normal. A value less than 3.6 mmol/L was considered hypokalemia, while a value greater than 5.5 mmol/L was considered hyperkalemia. Severe pneumonia was defined according to IMNCI guidelines.

The study included children meeting one or more of the following criteria: tachypnea, infants aged two months to one year with a respiratory rate exceeding 50 breaths/minute, children aged one to five years with a respiratory rate surpassing 40 breaths/minute, chest indrawing, wheezing, any general danger signs, such as the child's ability to drink or breastfeed, vomiting everything ingested, a history or complaint of convulsions, lethargy or unconsciousness, and stridor in a calm child.

A detailed history was obtained from the parents/guardians, relevant to the case, and a comprehensive clinical examination was conducted according to the proforma. Observations included chest recession, nasal flaring, cyanosis, grunting respiration, poor feeding, capillary refill time >2 seconds, and oxygen saturation <92%. Additionally, the status of nutrition and immunization was assessed.

At the time of admission, venous blood samples were collected without squeezing. Venous blood samples were used to estimate dyseletrolytemia (serum Na* and K*). The second venous sample was collected on the third day after admission and was used for the follow-up for dyselectrolytemia. The estimation of serum electrolytes was performed using either the Roche 9180 electrolyte analyzer (Roche Diagnostics, Indianapolis, IN) or the Diamond Diagnostics SmartLyte Electrolyte Analyzer (Diamond Diagnostics, Holliston, MA), employing the ion-selective electrode method. A chest X-ray and ECG were conducted in patients with hyperkalemia and also in patients with hypokalemia.

The correction of electrolytes was noted. The need for ICU admission and mechanical ventilation, along with outcomes and the duration of hospital stay (in days), was recorded. In our study, prolonged hospital stay is defined as a duration of hospital stay exceeding seven days.

Statistical analysis

Data analysis was conducted using SPSS Statistics for Windows, Version 17. (SPSS Inc., Chicago). The collected data were analyzed using frequencies and percentages.

## Results

In our study group the majority, accounting for 27 (33.8%) cases, belonged to the two months to one-year-old age group, with a male-to-female ratio of 1.9:1. Out of 80 children, boys accounted for 52 (65%), while 28 (35%) were girls. Severe pneumonia was more common in males (65%) than females. Both severe acute malnutrition and moderate acute malnutrition were found in 12 children, each accounting for 15% of the study population. No malnutrition was found in 56 patients (70%).

In this study group, dyselectrolytemia was present in 47 (59%) and absent in 33 (41%) patients (Table 1). The mean serum potassium level was less (*P*-value = 0.12) on day 3 (3.97 ± 0.25 mEq/L) as compared to that on day 1 (4.12 ± 0.74 mEq/L). The mean serum sodium level significantly increased (*P*-value < 0.0001) on day 3 (139.16 ± 2.57 mmol/L) as compared to that on day 1 (135.43 ± 6.7 mmol/L).

**Table 1:** Distribution of participants according to dyselectrolytemia.

Dyselectrolytemia	Number of children, *n* (%)
Present	47 (59)
Absent	33 (41)
Total	80 (100)

Out of 80 children, the majority had hyponatremia (31, 39%), followed by hypernatremia (3, 4%), hypokalemia (12, 15%), and hyperkalemia (1, 1%) (Table 2).

**Table 2:** Distribution of patients based on electrolyte imbalance in the study group (n = 80).

Electrolyte imbalance	Number of children, *n* (%)
Hyponatremia	31 (39)
Hypernatremia	3 (4)
Hypokalemia	12 (15)
Hyperkalemia	1 (1)
Total	47 (59)

We further examined the association between the duration of hospital stay and dyselectrolytemia in our study group. Out of 80 children with severe pneumonia, 5 (12.5%) children with dyselectrolytemia had a hospital stay of less than or equal to seven days and 35 (87.5%) children had a hospital stay of more than seven days (Table 3).

**Table 3:** Distribution of association between duration of hospital stay and dyselectrolytemia.

Duration of hospital stay (days)	Dyselectrolytemia		*n* (%)
	Present	Absent	
≤7	9	25	34 (42)
>7	38	8	46 (58)
Total	47	33	80 (100)

We further investigated the association between ICU admission and outcomes in the study group. Out of 17 patients (21.25%) who were admitted to the ICU, only 4 (23.52% of ICU-admitted patients) had a fatal outcome (Table 4). In this study, out of 80 patients with severe pneumonia, 17 (21.25%) required ICU stay. Among them, 16 patients had dyselectrolytemia, 6 (7.55%) needed mechanical ventilation, and 4 (5%) deaths occurred.

**Table 4:** Distribution of association between ICU admission and outcome in the study group.

ICU admission	Outcome		*n* (%)
	Survived	Death	
Yes	13	4	17 (21)
No	63	0	63 (79)
Total	76	4	80 (100)

## Discussion

Hyponatremia is the most common electrolyte abnormality in hospitalized patients. It is frequently found in children having pneumonia. Sodium deficit or an excess of water can cause hyponatremia. However, several contributing factors such as a primary illness, impaired water excretion, impaired vasopressin release, redistribution of sodium and water, the use of hypotonic fluids, sickle cell syndrome, and various drugs may also play a role in the development of hyponatremia [11-13]. Various studies have been conducted to parse out the correlation between pneumonia in children with hyponatremia. Zilberberg et al. described that CAP and nosocomial pneumonia significantly contribute toward resource utilization in hospitals and morbidity [14].

In this study, out of 80 severe pneumonia patients, 47 (59%) had electrolyte dyselectrolytemia. Hyponatremia (serum sodium <135 mEq/L) was the most common electrolyte imbalance observed, and it was prevalent in critically ill children as well. Mild-to-moderate hyponatremia was found in 15% to 30% of hospitalized patients, while severe hyponatremia was found in 1% to 4% of hospitalized patients. According to a study by Zilberberg et al., 28% of patients with CAP had hyponatremia at hospital admission [15]. Wrotek and Jackowska [16] reported hyponatremia in 33.3% of patients, and Patil [17] reported it in 26% of patients.

Among electrolytes, hyponatremia was the most common (31, 39%) in our study, followed by hypokalemia (12, 15%), hypernatremia (3, 4%), and hyperkalemia (1, 1%). Similarly, Singhi and Dhawan reported hyponatremia in 27%, hypernatremia in 3.7%, hypokalemia (K* <3.5 mEq/L) in 19%, and hyperkalemia (K* >6.5 mEq/L) in 2% in their study [10]. Sakellaropoulou et al. found that 64.8% of children with pneumonia had normal values of sodium at admission, 33.3% had mild hyponatremia, and 1.9% had moderate hyponatremia [18].

Hyponatremia is associated with a greater risk of death and increased length of hospital stay. In this study, dyselectrolytemia was present in 47 (59%) patients, with 35 (87.5%) patients staying for more than seven days and 5 (12.5%) patients staying for less than or equal to seven days. This relationship was clinically significant. In the present study, out of 80 patients with severe pneumonia, 17 (21.25%) needed ICU stay, of which 16 had dyselectrolytemia. Additionally, 6 (7.55%) patients needed mechanical ventilation, and 4 (5%) deaths occurred. Wrotek and Jackowska reported that hyponatremic patients had a longer duration of hospitalization in comparison with nonhyponatremic children [16]. However, Sakellaropoulou et al. reported a negative association between the duration of hospitalization and the degree of hyponatremia [18].

In another study by Singh and Dhawan, patients with hyponatremia showed 60% longer hospital stays. The same study also attributed hyponatremia to several conditions, including a twofold increase in complications along with 3.5 times higher mortality compared to that of normonatremia [12]. The severity of the aforementioned conditions increased in cases where hypokalemia and hyponatremia coexisted. Rahul and Jose [19] detected a statistically significant relationship between hyponatremia at admission and increased ICU admissions (17.9%, *P = *0.000) and an increased number of days (>2 days) in the ICU (*P *= 0.001). Patil also found a statistically increased mean hospital stay of patients with hyponatremia with pneumonia (9.54 ± 2.63 days) compared to those patients with normonatremia with pneumonia (6.43 ± 1.16 days) [17].

The major limitation of this study is the small sample size as this was done in a single center. Follow-up studies with larger sample sizes will provide more clarity and knowledge toward developing better management and therapeutic strategies.

## Conclusions

In this study, we observed that electrolyte imbalance in general, and hyponatremia in particular, is associated with an increased length of hospital stay and, in certain cases, a higher risk for death. Proper nutritional advice for children with pneumonia may decrease morbidity and mortality. The management of hyponatremia depends on the time of recognition, its onset, magnitude and severity, and associated risk factors, especially concerning neurological complications. Early detection and treatment of electrolyte imbalances in pneumonia cases will reduce prolonged hospital stays, ICU admissions, and the need for mechanical ventilation, ultimately contributing to a decrease in morbidity and mortality.
